# Subgrouping Poor Sleep Quality in Community-Dwelling Older Adults with Latent Class Analysis - The Yilan Study, Taiwan

**DOI:** 10.1038/s41598-020-62374-4

**Published:** 2020-03-25

**Authors:** Hsi-Chung Chen, Nai-Wei Hsu, Pesus Chou

**Affiliations:** 1 Department of Psychiatry & Center of Sleep Disorders, National Taiwan University Hospital, Taipei, Taiwan; 2 Division of Cardiology, Department of Internal Medicine & Community Medicine Center, National Yang-Ming University Hospital, Yilan, Taiwan; 3 Faculty of Medicine, School of Medicine, National Yang-Ming University, Taipei, Taiwan; 4 Public Health Bureau, Yilan County, Taiwan; 5 Community Medicine Research Center & Institute of Public Health, National Yang-Ming University, Taipei, Taiwan

**Keywords:** Psychiatric disorders, Geriatrics, Epidemiology

## Abstract

The manifestation of older adults with poor sleep quality is heterogeneous. Using data-driven classifying methods, the study aims to subgroup community-dwelling older adults with poor sleep quality. Adults aged 65 and older participated in the Yilan study. Poor sleep quality was defined using the Pittsburgh Sleep Quality Index. Latent class analysis with the 7 subscores of the Pittsburgh Sleep Quality Index as the indicators was used to generate empirical subgroups. Differences in comorbidity patterns between subgroups were compared. A total of 2622 individuals, of which 1011 (38.6%) had Pittsburgh Sleep Quality Index -defined poor sleep quality, participated. Three groups for poor sleep quality were specified in the latent class analysis: **High Insomnia** (n = 191, 7.3%), **Mild Insomnia** (n = 574, 21.9%), and **High Hypnotics** (n = 246, 9.4%). The **High Insomnia** and **Mild Insomnia** groups shared similar profiles but different severities in the 7 domains of the Pittsburgh Sleep Quality Index. In contrast, the **High Hypnotics** group had the lowest Pittsburgh Sleep Quality Index total scores and insomnia severity but had similar mental and physical comorbid patterns as the **High Insomnia** group. This finding suggests that poor sleep quality in community-dwelling older adults had various feature-based subgroups. It also implicates the development of group-centered interventions.

## Introduction

Sleep quality deteriorates along with aging, and it is partly related to the normal aging process and is a significant result of ill health^1^. Accordingly, poor sleep quality is prevalent in the older population^2^, and is related to various adverse health outcomes^3,4^. In particular, older adults with poor sleep quality have higher utilization of medical services and more mental and physical comorbidities than those with only night insomnia symptoms but without daytime repercussions^5^. Thus, identifying older adults with poor sleep quality and providing relevant intervention is important. However, the main attributes of poor sleep quality highly vary in different characteristic populations, a one-size-fits-all approach will not satisfy the multifactorial features of poor sleep quality. Therefore, subgrouping older adults with poor sleep quality may be capable of suggesting specific interventions, including in the community setting.

Presently, the Pittsburgh Sleep Quality Index (PSQI) is the most commonly used clinimetric instrument to measure sleep quality. The PSQI reflects several dimensions of sleep quality, and its global score is used to quantify and compare sleep quality in various populations^6^. The subscores of the PSQI provide further information for individual evaluation in clinical settings, and accordingly, clinicians could formulate specific sleep-related pathology and prescribe relevant interventions. The PSQI-defined poor sleep quality has been demonstrated to have multiple factor structures across various populations^7–10^, including the older population^11^. Furthermore, differently from other dimensions which showed a stable factor loading structure, the factor loading structure of the ‘sleep medication use’ varied in these studies^12^. Although hypnotics treatment for insomnia could alleviate insomnia symptoms, it may not consequently improve subjective sleep quality^13,14^. These evidences suggest that numerous clusters of individuals with different manifestations of sleep disturbances and sleep-related pathological mechanisms are included in the PSQI-defined poor sleep quality group. Besides, the pattern of hypnotics use may influence the classification of clusters. Thus, subgrouping the PSQI-defined poor sleep quality individuals is warranted to tailor specific interventions.

Latent class analysis (LCA) is a type of clustering analysis that has been widely used in sleep-related studies to subgroup changes in sleep duration^15^, sleep difficulty^16^, and insomnia symptoms^17^. In the older population, LCA is also used to classify changes in sleep quality^18^, the relationship between sleep disturbance and mortality risk^19^, and the relationship between activity rhythm and depressive symptoms^20^. Similar to other methods of cluster analyses, LCA intends to identify a latent variable that could explain the association between a set of variables. Traditional factor analysis examines the between-variable association, but LCA elucidates the between-individual relationship. By using LCA to subgroup patients with sleep disturbances, types and frequency of subjective symptoms, functional impairment (physical, emotional and cognitive dysfunction), and comorbid conditions (physical and psychiatric comorbidities) have been identified as important elements in distinguishing groups with sleep problems^21^. However, to the best of our knowledge, no studies have used LCA to classify the PSQI-defined poor sleep quality in large-scale, community-dwelling older adults with poor sleep quality.

Therefore, this study intended to determine whether LCA can subgroup community-dwelling older adults with a similar profile of poor sleep quality and examine whether an overarching variable can explain the sleep disturbance patterns. The validity and clinical significance of feature-based clusters were also examined by several external validators to compare the between-cluster differences in terms of sleep-wake related symptoms, the use of medical resources, comorbid mental and physical conditions, physical function, physical disability, and the quality of life.

## Methods

### Participants

The present study is a part of the Yilan study, which aimed to investigate the mental and physical health of community-dwelling older adults in Yilan City, Taiwan. The study design has been detailed previously^22^. Briefly, from August 2013 to November 2016, individuals aged ≥65 years living in Yilan City were randomly selected to participate in this study. Well-trained research assistants interviewed eligible participants face-to-face. Individuals who failed to provide a past medical history, could not complete the interview, or were unable to cooperate with the collection of anthropometric data because of physical disability or compromised cognitive function were excluded from this study. Finally, a total of 2622 older adults were included in this study. All participants provided written informed consent, and the Institutional Review Board of the National Yang-Ming University approved this study. All methods were performed in accordance with the relevant guidelines and regulations.

### Scoring and measuring the PSQI-defined poor sleep quality

Poor sleep quality was measured and defined using the PSQI. The PSQI is used to measure the overall sleep quality of individuals in one month. This index comprises 19 items that evaluate 7 components of sleep quality, including subjective sleep quality (component 1, C1), sleep onset latency (component 2, C2), total sleep duration (component 3, C3), sleep efficiency (component 4, C4), sleep disturbances (component 5, C5), use of sleep medication (component 6, C6), and daytime dysfunction (component 7, C7). The subscore of each component ranges from 0 to 3, and the maximum total composite score of the PSQI is 21. The cutoff score for PSQI-defined cases of poor sleep quality is 6 or more^6^. A Chinese version of the PSQI has been validated with adequate reliability^23^.

### External validators for verifying LCA-classified subgroups in the Yilan study

In the present study, several validators were used to examine the validity of LCA-classified subgroups. For the physical comorbidities, a history of diabetes mellitus, hypertension, heart diseases, hyperlipidemia, stroke, cancer, or gout was investigated. Medical diseases were recorded as present diseases only for participants who reported both diagnosis and treatment experiences^24^. The total number of medical morbidities was calculated. Along with the number of outpatient service visits in the past year, these two indices were used as the measurements of disease burden. The Groningen Activity Restriction Scale (GARS) was utilized to assess physical disability^25^. The GARS is a one-dimensional hierarchical scale used to grade the difficulties that a person might experience when performing activities of daily living without assistance. The total score (range, 18–72) provides a measure of the respondents’ ability to care for themselves and perform household activities. Handgrip strength (kg) was estimated using a dynamometer (Jamar, Jackson, MI, USA). Participants completed two trials for each hand, and the best performance of each hand was averaged as the final estimate of handgrip strength. The Short Form 12 Health Survey Version 2 (SF-12) was used to evaluate the health-related quality of life. The mental component summary (MCS) and physical component summary (PCS) comprising 12 items were used to measure the participant’s mental and physical function status in the past four weeks. The Chinese version of the SF-12 has been proven to be valid^26^. The Hospital Anxiety and Depression Scale (HADS) was utilized to measure the severity of depression and anxiety symptoms (score range of each subscale: 0–21)^27^. With respect to sleep-wake related parameters, the Chinese version of the 5-item Athens Insomnia Scale (AIS-5) was used to assess the severity of sleep complaints (score range: 0–15)^28^. The Chinese version of the Epworth Sleepiness Scale (ESS) was used to evaluate excessive daytime sleepiness (score range: 0–24)^29^.

### Other covariates

Sociodemographic data, including age, level of education, and living status (living alone vs. living with others) were obtained. Age was dichotomously categorized according to the definition of the young-old (<75 years) and old-old (≥75), repsectively^30^. In addition, information about the history of smoking, alcohol consumption, and exercise frequency in the past year and body height and weight data were collected. Body mass indexes of less than 18.5 kg/m^2^ and more than 24.0 kg/m^2^ were used to define underweight and overweight in older adults, respectively^31^.

### Statistical analyses

All statistical analyses were performed using SPSS for Windows, version 13.0 (SPSS Inc., Chicago, IL, USA) or the Mplus, version 7 (Muthen & Muthen) for LCA^32^. Univariate analyses were conducted using the χ^2^ test, *t*-test, or analysis of variance (ANOVA) test. In addition to LCA, other multivariate analyses were performed by general linear models. A *p-value* < 0.05 was considered statistically significant.

#### Data-driven subgrouping by LCA

In this study, LCA was used for classifying subgroups. Compared with traditional regression models, LCA has several advantages. First, LCA assumes neither linear relations between variables nor normal distributions for main variables or error terms, which are often violated in the real world. Moreover, LCA could analyze data in an exploratory fashion; thus, the number and type of latent classes are not known a priori^21^. In the present study, the parameters of LCA were estimated using the maximum likelihood estimation with robust standard errors. The 7 subscores of the PSQI, which were regarded as categorical variables, were used as the indicator variables for LCA. Before performing LCA, the case number allocated in each category of the respective subscore was examined. If the percentage of individuals in a specific category was less than 3%, it would be added into the adjacent category. To determine the number of latent classes needed in the present study, we used five tests as follows: (a) Akaike’s information criterion (AIC)^33^ and sample-size adjusted Bayesian’s information criterion (sBIC)^34^. Lower values for these criteria indicate superior model fits; (b) Entropy: a measure of the number of ways in which a system or data may be arranged. A value close to 1 indicates a clear classification^35^, and an average posterior probability of >0.7 suggests a clear and appropriate classification^36^, (c) Lo–Mendell–Rubin adjusted likelihood ratio test and parametric bootstrapped likelihood ratio test. These two tests are used to compare between k-class and k-1 class solution. A significant *p-value* for a test indicates a superior fit to a k class than to a k-1 class model^37^.

#### Examination of between-group differences among the LCA-defined clusters

After classifying feature-based subgroups in the Yilan cohort using LCA, the distinction between the 7 subscores of the PSQI across the subgroups was examined using ANOVA. Additional variables were used as external validators to examine the uniqueness of each class. Variations on these variables suggest that the clusters identified by LCA reflect distinct groups of older adults who have different clinical characteristics and sleep patterns. General linear models were used to discern the variations of external validators among the clusters. In the general linear model, multiple comparisons were corrected using the Bonferroni method.

## Results

Table 1 summarizes and compares the differences in sociodemographic, lifestyle, and clinical characteristics between older adults with fair and poor sleep quality. A total of 2662 community-dwelling older adults participated in the study; among them, 1011 (38.6%) had PSQI-defined poor sleep quality. Those with poor sleep quality were older (p = 0.04), were mostly female (p < 0.001), exercised less (p < 0.001), and had higher fall experience (p = 0.003) than those with fair sleep quality. Older adults with poor sleep quality had greater total and 7 subscores of the PSQI (all comparisons, p < 0.001), more severe insomnia (p < 0.001), and higher frequency of hypnotics use (p < 0.001) than those with fair sleep quality. However, no significant difference was found in daytime sleepiness. In addition, those with poor sleep quality had more adverse mental and physical conditions, including a number of physical diseases (p = 0.001), depression (p < 0.001), anxiety (p < 0.001), poor PCS (p < 0.001) and MCS (p < 0.001) of SF-12, great physical disability (p < 0.001), and weak handgrip strength (p < 0.001).

**Table 1:** The comparison of demographic and clinical characteristics of participants by sleep quality (n = 2622).

	Total	Pittsburgh Sleep Quality Index		*p-*value for χ^2^/t-test
		<6 (n = 1611)	≥ 6 (n = 1011)	
Age (years, mean ± SD)	76.6 ± 6.4	76.4 ± 6.6	76.9 ± 6.1	p = 0.04
Sex (n, %)				
Female	1548 (59.0)	850 (52.8)	698 (69.0)	p < 0.001
Education status (n, %)				
High school and above	508 (19.4)	375 (23.3)	133 (13.2)	p < 0.001
Middle school	322 (12.3)	207 (12.8)	115 (11.4)	
Primary school	1199 (45.7)	723 (44.9)	476 (18.2)	
Illiterate	593 (22.6)	306 (19.0)	287 (28.4)	
Body mass index (n, %)				
<18.5	105 (4.0)	64 (4.0)	41 (4.1)	p = 0.003
18.5–23.9	961 (36.7)	594 (36.9)	367 (36.3)	
≥24	1404 (53.5)	881 (54.7)	523 (51.7)	
Missing	152 (5.8)	72 (4.5)	80 (7.9)	
Living status (n, %)				
Alone	197 (7.5)	120 (7.4)	77 (7.6)	p = 0.88
Smoking status (n, %)				
Current smoker	238 (9.1)	164 (10.2)	74 (7.3)	p = 0.001
Ex-smoker	421 (16.1)	282 (17.5)	139 (13.7)	
Non-smoker	1963 (74.9)	1165 (72.3)	798 (78.9)	
Drinking status (n, %)				
Current drinker	2154 (82.2)	228 (14.2)	103 (10.2)	p = 0.002
Ex-drinker	137 (5.2)	93 (5.8)	44 (4.4)	
Non-drinker	331 (12.6)	1290 (80.1)	864 (85.5)	
Frequency of exercise (n, %)				
<3/week	1174 (44.8)	665 (41.3)	509 (50.3)	p < 0.001
Visits of outpatient service in 1 year (n, %)				
<1/month	2040 (77.8)	1280 (79.5)	760 (75.2)	p = 0.02
1–2/ month	494 (18.8)	285 (17.7)	209 (20.7)	
≥3/ month	87 (3.3)	45 (2.8)	42 (4.2)	
Falls in the past one year (n, %)	454 (17.3)	248 (15.4)	206 (20.4)	p = 0.001
Pittsburgh Sleep Quality Index (mean ± SD)	5.1 ± 4.0	2.5 ± 1.5	9.4 ± 2.9	p < 0.001
Athens Insomnia Scale (mean ± SD)	2.1 ± 3.9	0.3 ± 1.2	5.0 ± 4.8	p < 0.001
Insomnia (n, %)	560 (21.4)	40 (2.5)	520 (51.5)	p < 0.001
Epworth Sleepiness Scale (mean ± SD)	4.1 ± 4.8	4.1 ± 4.8	4.1 ± 4.8	p = 0.98
Hypnotics use (days, mean ± SD)	6.1 ± 11.7	2.3 ± 7.8	12.2 ± 14.3	p < 0.001
Number of physical diseases (mean ± SD)	1.6 ± 1.3	1.5 ± 1.2	1.7 ± 1.3	p < 0.001
Hospital Anxiety and Depression Scale (mean ± SD)				
Depression subscale	1.9 ± 2.6	1.5 ± 2.2	2.6 ± 3.0	p < 0.001
Anxiety subscale	2.3 ± 2.9	1.7 ± 2.3	3.3 ± 3.3	p < 0.001
Short-form 12 (mean ± SD)				
Mental health composite scores	58.4 ± 7.5	59.7 ± 6.2	56.3 ± 8.9	p < 0.001
Physical health composite scores	46.6 ± 9.3	48.3 ± 8.6	43.9 ± 9.8	p < 0.001
Groningen Activity Restriction Scale (mean ± SD)	23.0 ± 11.3	21.7 ± 9.7	25.1 ± 13.2	p < 0.001
Hand grip strength (kg, mean ± SD)	19.5 ± 8.5	20.5 ± 8.5	17.9 ± 8.1	p < 0.001

Table 2 summarizes the model fitness across 2–4 classes. The differences in AIC and sBIC between 3 and 4 classes were minimal, and the 3-class model had acceptable entropy (0.73). Furthermore, the 3-class model best fit the data in the Lo-Mendell-Rubin adjusted likelihood ratio test. In the parametric bootstrapped likelihood ratio test, the higher-class model consistently had a better fit than the lower-class model. Although the 4-class model also had a good model fit, the 3-class model was selected to be the best model considering its clinical significance and practicality in community medicine.

**Table 2:** Model fitting results for 2- to 4-class solutions in the latent class analysis (n = 1011).

	2 classes	3 classes	4 classes
Akaike Information Criterion	14225.28	13949.14	13883.87
Sample-Size Adjusted Bayesian Information Criterion	14289.75	14046.73	14014.56
Entropy	0.77	0.73	0.81
Lo-Mendell-Rubin Adjusted Likelihood Ratio test	p < 0.001	p = 0.0002	p = 0.13
Parametric Bootstrapped Likelihood Ratio test	p < 0.001	p < 0.001	p < 0.001

**Figure 1:**
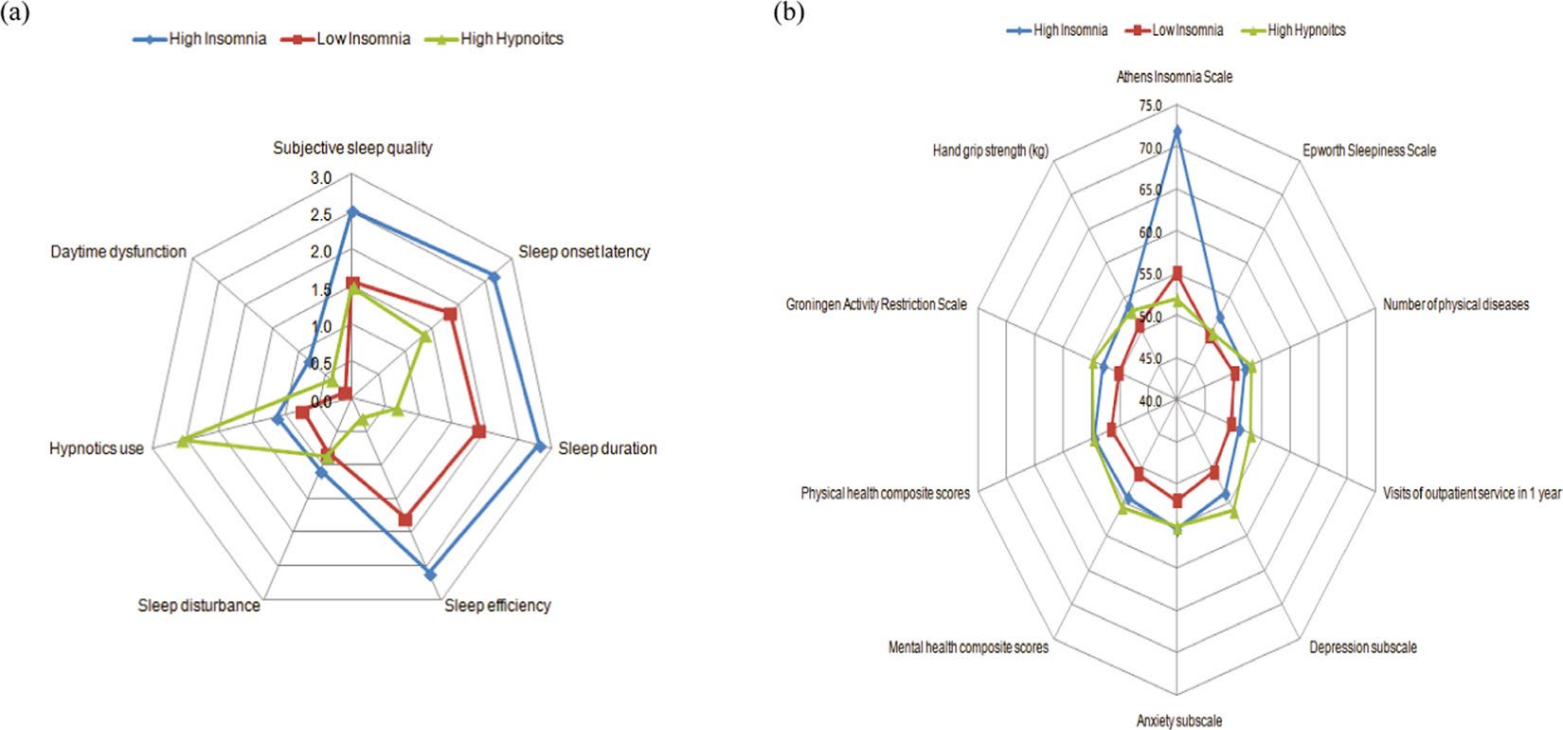
Radar charts comparing profile patterns among empirically-derived subgroups of older adults with Pittsburgh Sleep Quality Index (PSQI)-defined poor sleepers. Panel (a) the comparisons of 7-subscores of PSQI, Panel (b) the comparisons for various external validators. All scores of external validators were transformed to t-scores, by which the higher scores indicated worse health status.

According to the LCA results and distribution of the subscores in the PSQI, the 3 subgroups were designated as *high insomnia* (HI, n = 191), *mild insomnia* (MI, n = 574), and *high hypnotics* (HH, n = 246). The panel (a) in Fig. 1 profiles the pattern of the 7 subscores of the PSQI across the 3 subgroups. Participants in the HI group had the most severe night insomnia symptoms and daytime dysfunction. Participants in the HH group had the lowest scores on the night insomnia symptoms among those in the three groups, but they had higher daytime dysfunction than those in the MI group. Participants in the MI group had a moderate severity of night insomnia symptoms compared with those in the other two groups, but they had the least use of hypnotic and minimal daytime dysfunction among those in the three groups. In general, participants in the HI and MI groups had similar configurations over the 7 domains of the PSQI but differed in the severity of each domain. In contrast, participants in the HH group had mild insomnia symptoms, but they were characterized by frequent hypnotics use and residual daytime dysfunction despite the use of sleeping pills. The linear relationship between the subscore of the C6 (hypnotics use) and other subscores was found across the MI and HI groups. However, a non-linear pattern between the frequency of hypnotics use and the other domains was observed in the HH group. Notably, participants in the group with the most frequent hypnotic use had the lowest average C1–C4 subscores but the moderate daytime dysfunction subscore among those in the 3 subgroups.

Table 3 compares the composite scores and 7 subscores of the PSQI between the fair sleep quality group and the 3 LCA-specified poor sleep quality subgroups. The composite scores and the other subscores, except for those of the components of subjective sleep quality and sleep disturbance in the MI and HH groups, significantly differed among the 3 LCA-specified subgroups. Older adults in the HI and MI groups had consistently higher composite scores and 7 subscores than those with fair sleep quality. Interestingly, although the composite scores and most of the subscores of participants in the HH group remained higher than those of the individuals with fair sleep quality, the components of sleep duration and habitual sleep efficiency showed no significant differences.

**Table 3:** Dimensional comparison of sleep quality by empirically-derived subgroups.

	(a) Pittsburgh Sleep Quality Index <6 (n = 1611, 61.4%)	Pittsburgh Sleep Quality Index ≥6			Omnibus *p*-value for ANOVA
		(b) High insomnia (n = 191, 7.3%)	(c) Mild insomnia (n = 574, 21.9%)	(d) High hypnotics (n = 246, 9.4%)	
	Mean (SD)	Mean (SD)	Mean (SD)	Mean (SD)	
Total scores	2.46 (1.54)^b,c,d,#^	13.62 (2.16)^a,c,d^	8.77 (2.21)^a,b,d^	7.61 (1.68)^a,b,c^	p < 0.001
Subjective sleep quality	0.79 (0.50)^b,c,d^	2.52 (0.50)^a,c,d^	1.55 (0.52)^a,b^	1.46 (0.60)^a,b^	p < 0.001
Sleep latency	0.32 (0.61)^b,c,d^	2.64 (0.69)^a,c,d^	1.83 (1.06)^a,b,d^	1.35 (0.99)^a,b,c^	p < 0.001
Sleep duration	0.56 (0.77)^b,c^	2.81 (0.45)^a,c,d^	1.91 (0.87)^a,b,d^	0.67 (0.87)^b,c^	p < 0.001
Habitual sleep efficiency	0.23 (0.52)^b,c^	2.61 (0.91)^a,c,d^	1.79 (0.98)^a,b,d^	0.30 (0.53)^b,c^	p < 0.001
Sleep disturbances	0.38 (0.49)^b,c,d^	1.09 (0.43)^a,c,d^	0.82 (0.39)^a,b^	0.87 (0.53)^a,b^	p < 0.001
Use of sleep medication	0.15 (0.62)^b,c,d^	1.13 (1.38)^a,c,d^	0.75 (1.26)^a,b,d^	2.57 (0.96)^a,b,c^	p < 0.001
Daytime dysfunction	0.03 (0.19)^b,c,d^	0.82 (0.76)^a,c,d^	0.13 (0.37)^a,b,d^	0.40 (0.65)^a,b,c^	p < 0.001

^#^Each group was labeled as a to d, respectively. Superscript letters denoted significant between-group differences in post-hoc comparisons.

Table 4 compares the differences of the external validators between older adults with fair sleep quality and those in the 3 LCA-specified subgroups. Among the 3 clusters of older adults with poor sleep quality, the HI group had the greatest AIS-5 scores, but the HH group had the lowest AIS-5 scores. Although the scores on the HADS subscale were higher in the HH group, those on all the other validators did not significantly differ between the HI and HH groups. Notably, the extent at which the HI and HH groups differed from the MI group seemed subtle. Specifically, daytime sleepiness characterized the difference between the HI and MI groups. In addition, adverse physical conditions, including a higher number of physical diseases, more frequent use of outpatient services, worse PCS, higher scores on GARS, and weaker handgrip strength were different between the HH and MI groups. In contrast, no significant difference was observed between old adults in the MI group and those who had fair sleep quality in terms of the number of outpatient services used, physical disability, and handgrip strength. These findings suggest that older adults in the HI and MI groups share similar patterns but have different severity in the profile of validators, in which adverse mental conditions characterize these two subgroups. In contrast, in addition to adverse mental conditions, more unfavorable physical conditions were found in the HH group. The panel (b) in Fig. 1 depicts the distribution pattern of external validators; in general, the geometric shape of the HI group is a scaled-up version of the MI group. In contrast, the HH group had the lowest insomnia severity among the three groups, but the HH and HI groups had similar levels of adverse mental/physical conditions.

**Table 4:** General linear models for the comparison of clinical outcomes between good sleepers and subgroups of participants with poor sleep quality*.

	(a) Pittsburgh Sleep Quality Index <6^ǂ^ (n = 1611, 61.4%)	Pittsburgh Sleep Quality Index ≥6		
		(b) High insomnia (n = 191, 7.3%)	(c) Mild insomnia (n = 574, 21.9%)	(d) High hypnotics (n = 246, 9.4%)
	Estimated mean (95% CI)	Estimated mean (95% CI)	Estimated mean (95% CI)	Estimated mean (95% CI)
Athens Insomnia Scale	0.29 (0.16–0.42)^b,c,d^	10.49 (10.12–10.86)^a,c,d^	3.98 (3.76–4.19)^a,b,d^	2.80 (2.48–3.12)^a,b,c^
Epworth Sleepiness Scale	4.10 (3.87–4.33)	5.03 (4.35–5.71)^c^	3.81 (3.42–4.20)^b^	3.96 (3.36–4.55)
Number of physical diseases	1.51 (1.45–1.57)^b,d^	1.78 (1.60–1.96)^a^	1.60 (1.50–1.71)^d^	1.96 (1.80–2.11)^a,c^
Visits of outpatient service in 1 year	1.23 (1.21–1.26)^d^	1.31 (1.23–1.38)	1.24 (1.20–1.28)^d^	1.41 (1.35–1.47)^a,c^
Hospital Anxiety and Depression Scale				
Depression subscale	1.56 (1.44–1.68)^b,c,d^	2.63 (2.28–2.99)^a,c,d^	2.00 (1.79–2.20)^a,b,d^	3.42 (3.11–3.73)^a,b,c^
Anxiety subscale	1.78 (1.65–1.92)^b,c,d^	3.58 (3.20–3.96)^a,c^	2.74 (2.52–2.96)^a,b,d^	3.64 (3.31–3.98)^a,c^
Short-form 12				
Mental health composite scores	59.61 (59.26–59.97)^b,c,d^	55.54 (54.51–56.58)^a,c^	57.71 (57.12–58.31)^a,b,d^	54.31 (53.41–55.22)^a,c^
Physical health composite scores	47.86 (47.46–48.27)^b,c,d^	43.77 (42.60–44.94)^a^	43.45 (44.78–46.13)^a,d^	43.02 (42.00–44.05)^a,c^
Groningen Activity Restriction Scale	22.09 (21.60–22.58)^b,d^	25.08 (23.65–26.50)^a^	23.07 (22.26–23.89)^d^	27.45 (26.20–28.70)^a,c^
Hand grip strength (kg)	19.61 (19.30–19.92)^d^	18.71 (17.81–19.61)	19.82 (19.31–20.33)^d^	18.32 (17.52–19.11)^a,c^

^*^Covariates included age, sex, education, living status, exercise, smoking, alcohol drinking, body mass index and falls in the past year.

^ǂ^Each group was labeled as a to d, respectively. Superscript letters denoted significant between-group differences in post-hoc comparisons.

## Discussion

Using LCA, the present study successfully classified older adults with poor sleep quality into 3 feature-based clusters. These subgroups differed in the 7 domains of the PSQI. Variations on the external validators among these subgroups provided further evidence that these clusters represent distinct groups of older adults with differential sleep and clinical characteristics. To the best of our knowledge, this study is the first attempt to apply LCA to acquire empirical subgroups in a large-scale cohort of community-dwelling older adults with poor sleep quality.

The PSQI was hypothesized as a single construct when it was designed^6,38^. However, in the study of the older population, plural constructs have been observed. In older adults in the United States, the original 7 dimensions of the PSQI were found to embed 3 factors, including ***sleep quality*** (subjective sleep quality, sleep latency, and sleep medication use), ***sleep efficiency*** (sleep duration and habitual sleep efficiency), and ***daily disturbance*** (sleep disturbance and daytime dysfunction)^11^. In Asia, the 3 factors were also established in the older population, including ***sleep quality*** (subjective sleep quality and sleep latency), ***sleep efficiency*** (sleep duration and habitual sleep efficiency), and ***daily disturbance*** (sleep disturbance, sleeping medication use, and daytime dysfunction)^39^. Except for sleeping medication use, the other dimensions of the PSQI load in factor structures have similar patterns across these two studies. According to the profile of the 7 domains of the LCA-specified clusters in this study, the association between the subscores of sleep medication use and the other domains of the PSQI showed a non-linear pattern, which may partly explain why sleep medication use was occasionally found to load poorly into various factor structures in previous studies^12^.

In this study, compared with individuals with fair sleep quality, mean PSQI total score and each component subscore were higher in older adults who had PSQI-defined poor sleep quality. This finding is reasonable and consistent with the literature^12^. In contrast, the present study also illustrated the heterogeneity in the PSQI-defined poor sleep quality individuals. The HH group had lower total and sleep latency, sleep duration, and sleep efficiency component scores compared with the HI and MI groups. Furthermore, even the HH group had higher PSQI global scores than the one with fair sleep quality, the sleep duration and sleep efficiency in the HH group were comparable with the one with fair sleep quality. However, the HH group showed a worse daytime function, compared with both the PSQI-defined fair sleep quality and the MI groups. This finding echoes previous arguments that a frequent use of hypnotics might alleviate night insomnia symptoms but not necessarily produce a better daytime functioning^13^.

The PSQI has been found to have moderate to high correlation with insomnia^40^, poor health-related quality of life^8^, disability score^41^, depression^42^, and anxiety^42^. In addition, in-depth elucidation of the essence of poor sleep quality is important for the tailored intervention. The present study found greater sleep-related symptoms, more health care utilization, more physical and mental comorbidities, poorer health-related quality of life, more disability, and poorer functional capacity in LCA-specified subgroups than in the fair sleep quality group, and the present findings are in line with those in previous studies. However, the profiles of impairment differed in types and magnitude across the 3 subgroups, suggesting that different etiologies and interventions are warranted. In the present study, participants in both the HI and HH groups had higher mental and physical impairments, but only those in the HI group had prominent daytime sleepiness, an impact of insomnia. In contrast, participants in the HH group had less insomnia-related symptoms but more early signs of frailty, such as weak muscle strength. In addition, participants in the HI and MI groups had almost similar patterns of sleep disturbance, but those in the MI group had lower severity of insomnia, as did those in the HH group. Thus, those who had the PSQI-defined poor sleep quality but scored low in the final composite scores could be classified as two different groups. One group included individuals with the real low insomnia (the MI, the average PSQI composite scores: 8.77 ± 2.21), and the other group included those with treated insomnia with frequent hypnotics use and high comorbidities (the HH, the average PSQI composite scores: 7.61 ± 1.68). The findings are in line with a previous suggestion that in addition to the total scores, other PSQI factors should be used by clinicians to prevent missing information about significant sleep impairment^11^. Moreover, the present study suggests that the differential presentation and combination of the 7 domains of the PSQI may have various pathological mechanisms and different impacts; therefore, specific evaluation and intervention are warranted.

Our findings have some implications for community medicine. Although guidelines for sleep disturbance have been established in older adults, it is not efficient and cost-effective to deliver interventions non-differentially, such as hypnotics or cognitive behavior therapy^43^. Cognitive behavior therapy comprises several modules that demand different levels of professions. All these considerations influence how to structure the proper intervention at the population level. For example, in the HH group, the control of insomnia symptoms was not equal to the adequate management of comorbid conditions, especially depression and the pre-frailty status. Thus, a detailed review of the adequacy of the treatment for all comorbid conditions and the physical capacity in the HH group is as important as the intuitive idea of hypnotic reduction. If the comorbidities have been well controlled, the clinicians can justify the dosage of hypnotics in these older adults with complex comorbid insomnia. Older adults in the HH group need a hospital-based setting to fulfill these requests for intervention. Furthermore, because of complicated comorbid conditions, a barrier-free referral to sleep specialists may be best for participants in this group. In contrast, the HI group included older adults with inadequate evaluation and treatment for insomnia. Therefore, a comprehensive evaluation with relevant intervention is essential for participants in this group. Referral to primary care physicians should be an ideal starting point. Finally, older adults in the MI group were characterized by mild insomnia symptoms and limited functional impairment. Basic non-pharmacological interventions, such as sleep hygiene, stimulus control therapy, and relaxation, may be delivered first in the community in group therapy. Furthermore, signs of exacerbation should be taught to adults in the MI group to prevent the development of chronic insomnia and ensure the optimal timing of self-referral to physicians.

The present study has several limitations. First, the generalizability of our findings to other cohorts or those who are institutionalized, cognitively compromised, or residing in rural areas is unknown. Second, although the clinical profiles of the 3 LCA-specified subgroups differed, the external validators used in the present study did not evaluate the core pathology of sleep disturbance, such as pre-sleep hyperarousability, maladaptive sleep-related behaviors, or dysfunctional attitude and behavior to sleep. Thus, the most optimal and efficient intervention for each subgroup is still uncertain. Thirdly, in the present study, we used a score equal to or greater than 6 (the original cutoff suggested by the PSQI developer) as the cutoff to define PSQI poor sleep quality^6^. In fact, the optimal cutoff of the PSQI has not been determined in community-dwelling older adults. Thus, the validity of the PSQI-defined poor sleep quality in this study may be compromised. However, community-dwelling older adults in Taiwan who scored 6 or more at the PSQI have been found to have a higher risk for various adverse health outcomes^4,22,44,45^. These evidences, at least partly, validates the clinical relevance to generalize the original cutoff to community older populations in Taiwan.

Because poor sleep quality is a prevalent and etiologically complex problem in the older adults, the development of an efficient and specific screen and intervention strategy is crucial for community-dwelling population. The present study used data-driven approach to cluster phenotypic homogeneity subgroups of older adults with poor sleep quality. These feature-based subgroups also illustrated differential clinical profiles. In the future, a short and valid instrument may be developed accordingly to quickly screen and classify these subgroups. Eventually, we could expect an efficient and precise group intervention for community-dwelling older adults with poor sleep quality.

## Data Availability

The datasets generated during and/or analysed during the current study are available from the corresponding author on reasonable request.
